# *Stellate* Genes and the piRNA Pathway in Speciation and Reproductive Isolation of *Drosophila melanogaster*

**DOI:** 10.3389/fgene.2020.610665

**Published:** 2021-01-22

**Authors:** Vladimir E. Adashev, Alexei A. Kotov, Sergei S. Bazylev, Aleksei S. Shatskikh, Alexei A. Aravin, Ludmila V. Olenina

**Affiliations:** ^1^Laboratory of Biochemical Genetics of Animals, Institute of Molecular Genetics, National Research Centre “Kurchatov Institute”, Moscow, Russia; ^2^Laboratory of Analysis of Clinical and Model Tumor Pathologies at the Organismal Level, Institute of Molecular Genetics, National Research Centre “Kurchatov Institute”, Moscow, Russia; ^3^Division of Biology and Biological Engineering, California Institute of Technology, Pasadena, CA, United States

**Keywords:** *Drosophila*, *Stellate* genes, piRNA pathway, reproductive isolation, hybrid sterility

## Abstract

One of the main conditions of the species splitting from a common precursor lineage is the prevention of a gene flow between diverging populations. The study of *Drosophila* interspecific hybrids allows to reconstruct the speciation mechanisms and to identify hybrid incompatibility factors that maintain post-zygotic reproductive isolation between closely related species. The regulation, evolution, and maintenance of the testis-specific *Ste-Su(Ste)* genetic system in *Drosophila melanogaster* is the subject of investigation worldwide. X-linked tandem testis-specific *Stellate* genes encode proteins homologous to the regulatory β-subunit of protein kinase CK2, but they are permanently repressed in wild-type flies by the piRNA pathway via piRNAs originating from the homologous Y-linked *Su(Ste)* locus. Derepression of *Stellate* genes caused by *Su(Ste)* piRNA biogenesis disruption leads to the accumulation of crystalline aggregates in spermatocytes, meiotic defects and male sterility. In this review we summarize current data about the origin, organization, evolution of the *Ste-Su(Ste)* system, and piRNA-dependent regulation of *Stellate* expression. The *Ste-Su(Ste)* system is fixed only in the *D. melanogaster* genome. According to our hypothesis, the acquisition of the *Ste-Su(Ste)* system by a part of the ancient fly population appears to be the causative factor of hybrid sterility in crosses of female flies with males that do not carry Y-linked *Su(Ste)* repeats. To support this scenario, we have directly demonstrated *Stellate* derepression and the corresponding meiotic disorders in the testes of interspecies hybrids between *D. melanogaster* and *D. mauritiana*. This finding embraces our hypothesis about the contribution of the *Ste-Su(Ste)* system and the piRNA pathway to the emergence of reproductive isolation of *D. melanogaster* lineage from initial species.

**Graphical Abstract:**
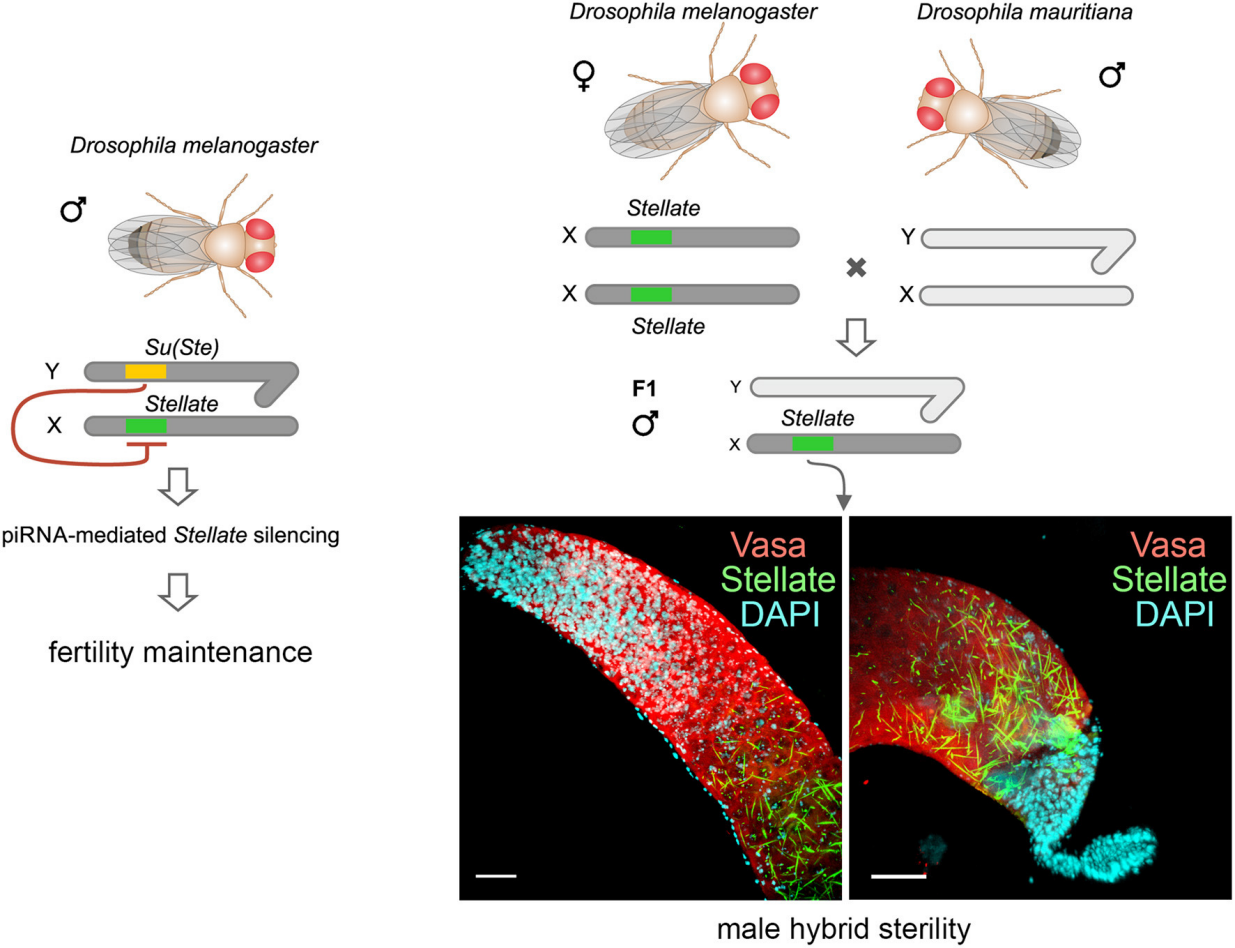
The *Stellate-Su(Ste)* genetic system is maintained only in the *D. melanogaster* genome. X-linked testis-specific *Stellate* genes are repressed in the testes of wild-type flies with the aid of piRNAs derived from the homologous Y-linked *Su(Ste)* locus that is essential for male fertility. Derepression of *Stellate* genes in the testes of hybrids of *D. melanogaster* with closely related *D. mauritiana* leads to the crystalline aggregate formation and causes strong meiotic failures and male hybrid sterility. Thus, the *Stellate-Su(Ste)* system and the piRNA pathway contribute to the emergence of the reproductive isolation of *D. melanogaster* from initial species.

## Introduction

At the beginning of the XXI century, a new class of small regulatory RNAs, piRNAs, was discovered in the testes of *Drosophila melanogaster* (Aravin et al., 2001, 2004; Vagin et al., 2006). X-linked *Stellate* genes, encoding proteins with homology to the regulatory β-subunit of protein kinase CK2, are currently known as the main targets of piRNA silencing in *D. melanogaster* testes (Nishida et al., 2007; Ryazansky et al., 2016; Kotov et al., 2019) and the maintenance of their repressed state is essential for male fertility (Palumbo et al., 1994; Bozzetti et al., 1995). *Stellate* genes are repressed via complementary piRNAs derived from transcripts of the homologous Y-linked *Suppressor of Stellate* [*Su(Ste)*] locus. The *Ste-Su(Ste)* family is present in the genome as abundant homologous tandem repeats found exclusively in *D. melanogaster* (Livak, 1984, 1990; Balakireva et al., 1992; Tulin et al., 1997). Loss of the *Su(Ste)* locus leads to *Stellate* derepression, accumulation of abundant crystalline aggregates in spermatocytes and to meiotic disorders (Hardy et al., 1984; Palumbo et al., 1994; Bozzetti et al., 1995). However, the biological functions of the *Ste-Su(Ste)* system have remained enigmatic for a long time. Currently, numerous data have been accumulated allowing the reconstruction of the essential stages of *Ste-Su(Ste)* evolution and piRNA-mediated regulation of *Stellate* gene expression. Our analysis involving interspecies hybrids reveals the contribution of the *Ste-Su(Ste)* system to hybrid male sterility. This is the first finding that the disruption of piRNA silencing of a protein-coding gene is able to cause the reproductive isolation of closely related species. In this review, we focus on analyzing the recent advances in understanding of the functional significance of the *Ste-Su(Ste)* genetic system and novel functions of piRNAs in reproductive isolation.

## Discovery of the *Crystal-Stellate* System and Their Structural Organization

The *crystal-Stellate* genetic system was discovered by studying the testes of *D. melanogaster* males with a missing Y chromosome (X/0) using the phase contrast microscopy. Crystalline aggregates of star-like and needle-like shape were found in the nuclei and cytoplasm of primary spermatocytes in these testes (Meyer et al., 1961). It was later shown that the testes of X/0 males also exhibited defects in the condensation and segregation of meiotic chromosomes, such as frequent chromosome non-disjunctions, and X/0 males were sterile (Lifschytz and Hareven, 1977; Hardy et al., 1984).

Now it is established that the *crystal-Stellate* genetic system contains several interacting loci mapping to the X and Y chromosomes. The Y chromosome of *D. melanogaster* is completely heterochromatic and contains only a few genes, mainly responsible for male fertility (Charlesworth, 2001; Hoskins et al., 2002; Chang and Larracuente, 2019). The first uncovered locus of the *crystal-Stellate* system was mapped to the h11 region of the mitotic prometaphase map of the Y chromosome. The loss of this locus or even its partial deletion was found to be sufficient for the crystal accumulation in spermatocytes (Hardy et al., 1984). Thus, the locus was named *crystal (cry)*, but later it was renamed to *Suppressor of Stellate* [*Su(Ste)*] (Hardy et al., 1984). Along with the generation of crystalline aggregates in the testes of males with a deficiency in the *cry* locus (*X/Y cry*^1^), similar defects of chromosome condensation and segregation with the *X/0* male testes were found (Palumbo et al., 1994).

The components of the system include two *Stellate (Ste)* loci, one of which resides in euchromatic cytolocation 12E1-2 of the X chromosome, whereas the other is mapped to pericentromeric heterochromatin of the X chromosome (the h26 region of the mitotic prometaphase map) (Hardy et al., 1984; Livak, 1984; Palumbo et al., 1994; Tulin et al., 1997). Molecular analysis revealed that the *crystal* and *Stellate* loci consist of multiple homologous tandemly repeated sequences (Livak, 1984, Figure 1). The severity of meiotic abnormalities, abundance and shape of crystals in the *cry*^1^ testes have been shown to depend on the *Ste* allele (Livak, 1984; Palumbo et al., 1994). The low-copy *Ste* + allele contains a small number of *Stellate* repeats (15–50 copies) and leads only to the appearance of little needle-like aggregates, weak meiotic disturbances and reduced male fertility, whereas the high-copy *Ste* allele (150–400 copies) leads to the formation of a multitude of crystals, visible under phase contrast as star-shaped entities, severe meiotic defects and complete sterility. Non-disjunction of the XY- and 2nd chromosomes, fragmented chromatin, and chromatin bridges have been found among the intrinsic meiotic defects in the testes of *cry*^1^ males. However, in the examined natural and laboratory lineages of *D. melanogaster*, the *Ste*+ alleles significantly predominate over the *Ste* ones (Palumbo et al., 1994). The severity of male fertility defects and the degree of meiotic disorders are associated with the number of *Stellate* copies and independent from the ratio of euchromatic and heterochromatic *Stellate* repeats. The boundary for fertility is considered to be 50–60 *Stellate* copies; the presence of more copies in the genome leads to complete male sterility (Palumbo et al., 1994). *Stellate* genes are expressed in the testes of *cry*^1^ males as 750 nt polyadenylated transcripts (Livak, 1990), and their abundance is proportional to the number of repeats in both *Stellate* loci (Palumbo et al., 1994). In the *cry*^1^ testes *Stellate* transcripts from both loci are translated generating small proteins of about 17–18 kDa, which have homology with the regulatory β-subunit of protein kinase CK2, CK2β (Livak, 1990; Bozzetti et al., 1995; Egorova et al., 2009; Olenkina et al., 2012b). Stellate proteins, products of the heterochromatic and euchromatic clusters, possess high intra-cluster homogeneity, having minor differences in amino acid sequence between themselves and slightly different electrophoretic mobility (Olenkina et al., 2012b). Immunostaining of the *cry*^1^ testes with anti-Stellate antibodies reveals that Stellate is main or the only component of crystalline aggregates (Bozzetti et al., 1995; Egorova et al., 2009, Figures 2A,D). In wild-type flies, *Stellate* gene expression is strongly suppressed and no Stellate proteins are detected (Figure 2B).

**Figure 1:**
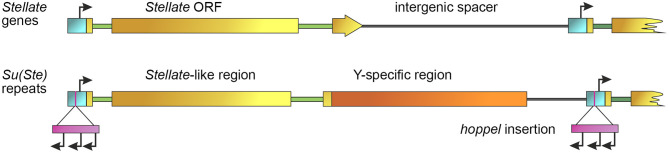
General scheme of *Stellate* and *Su(Ste)* repeats. Promoters are indicated by a blue color bar, introns are depicted by green lines, intergenic spacers are depicted by gray lines. *Stellate* gene contains an ORF (brown color bar) and two introns (green lines). An individual *Su(Ste)* repeat carries the region homologous to the *Stellate* ORF (brown color bar), Y-specific region (orange bar) and an insertion of the defective *hoppel* transposon (violet bar) flanked by inverted repeats (not shown) in the promoter. Start sites of sense transcription of *Stellates* and *Su(Ste)* and multiple starts of antisense *Su(Ste)* transcription within the body of *hoppel* are indicated by black arrows [modified from Aravin et al. (2001)].

**Figure 2:**
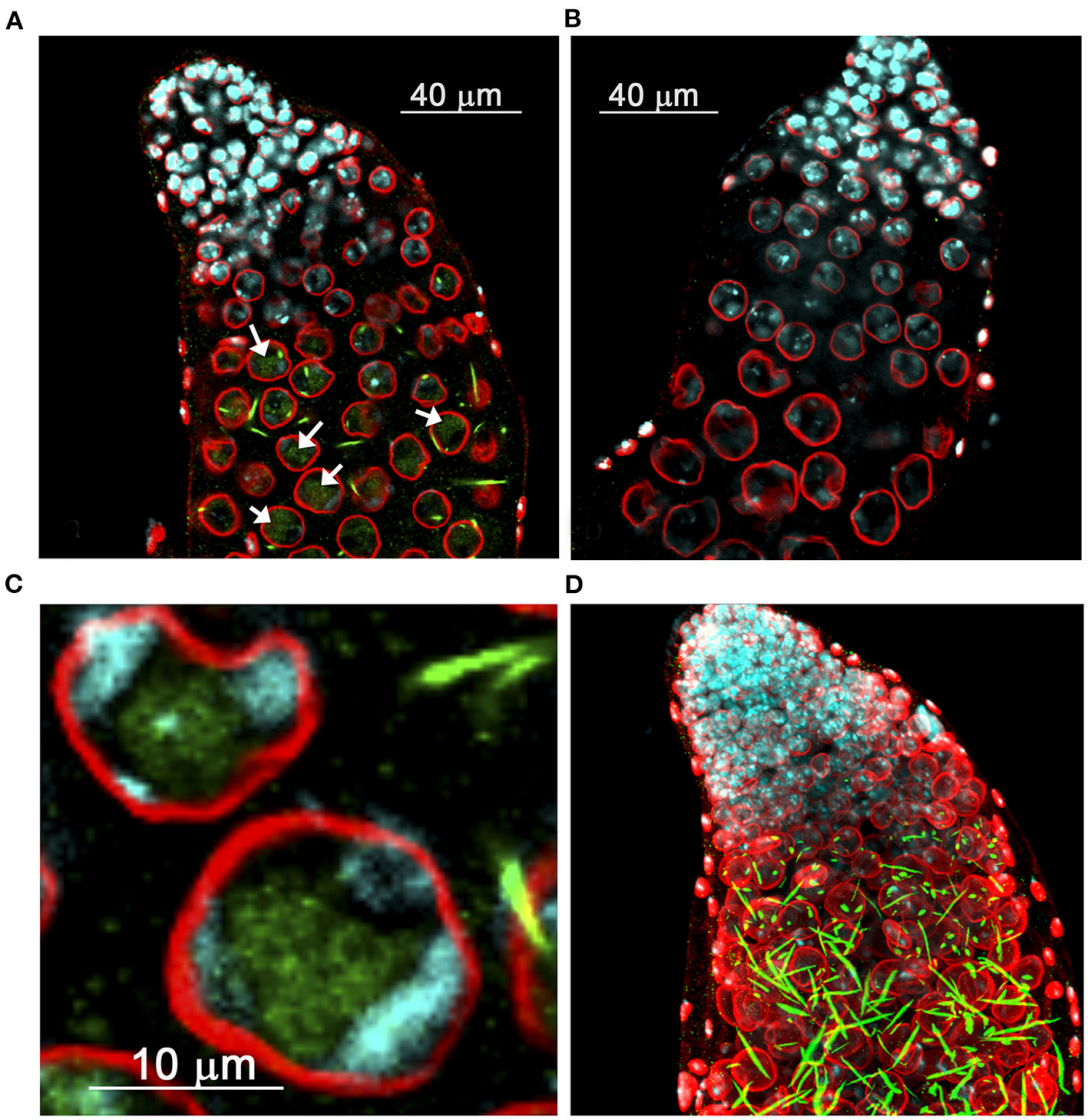
Distribution of derepressed Stellate protein in the testes of *D. melanogaster*. **(A–C)** Internal confocal slices of stained testis preparation of *cry*^1^ males **(A,C)** and wild-type control **(B)**. Testes were immunofluorescently stained with anti-Stellate (green) and anti-lamin (red) antibodies, chromatin was stained with DAPI (cyan). Anti-lamin staining indicates nuclear membrane position. **(A,C)** Diffuse Stellate signals in the nuclei (arrows in A) and bright needle-like and dot-like crystalline Stellate aggregates mainly in the cytoplasm are seen in spermatocytes of *cry*^1^ males. **(C)** The nuclei of mature spermatocytes. **(D)** 3D reconstruction of the stained testis preparation of *cry*^1^ males. **(A–C)** are reproduced from Figure 2 in Egorova et al. (2009). **(D)** is reproduced from Figure 2 in Kibanov et al. (2013) by permission of Elsevier (Licenses ## 4913121387410 and 4913131090753).

The organization of the *Su(Ste)* locus has also been studied in detail. According to previously published data in most laboratory strains of *D. melanogaster* the number of *Su(Ste)* repeats comprises about 80 copies, whereas in natural populations, strains with 240 repeats were found (Lyckegaard and Clark, 1989; Balakireva et al., 1992; McKee and Satter, 1996). However, recent Y chromosome assembly using the *Iso1* strain of *D. melanogaster* with improved annotation of both protein-coding genes and repeats contains more than 500 *Su(Ste)* copies (Chang and Larracuente, 2019). The size of a typical complete *Su(Ste)* repeat is about 28 000 nt. It consists of three main parts: the region homologous to *Stellate* gene, the *AT*-rich region specific for the Y chromosome, and the insertion of transposable element *hoppel* (*1360*) into the promoter sequence (Figure 1). *Su(Ste)* repeats are transcribed and processed to polyadenylated mRNAs, however, unlike *Stellate* transcripts, they contain numerous frameshift mutations due to point mutations and deletions (Balakireva et al., 1992; Shevelyov, 1992). Translation products of *Su(Ste)* repeats are not detected. The insertion of a defective transposon *hoppel* copy is responsible for antisense transcription of *Su(Ste)* repeats (Aravin et al., 2001).

## Reconstruction of *Ste-Su(Ste)* System Origin in the *D. Melanogaster* Genome

**Figure 3:**
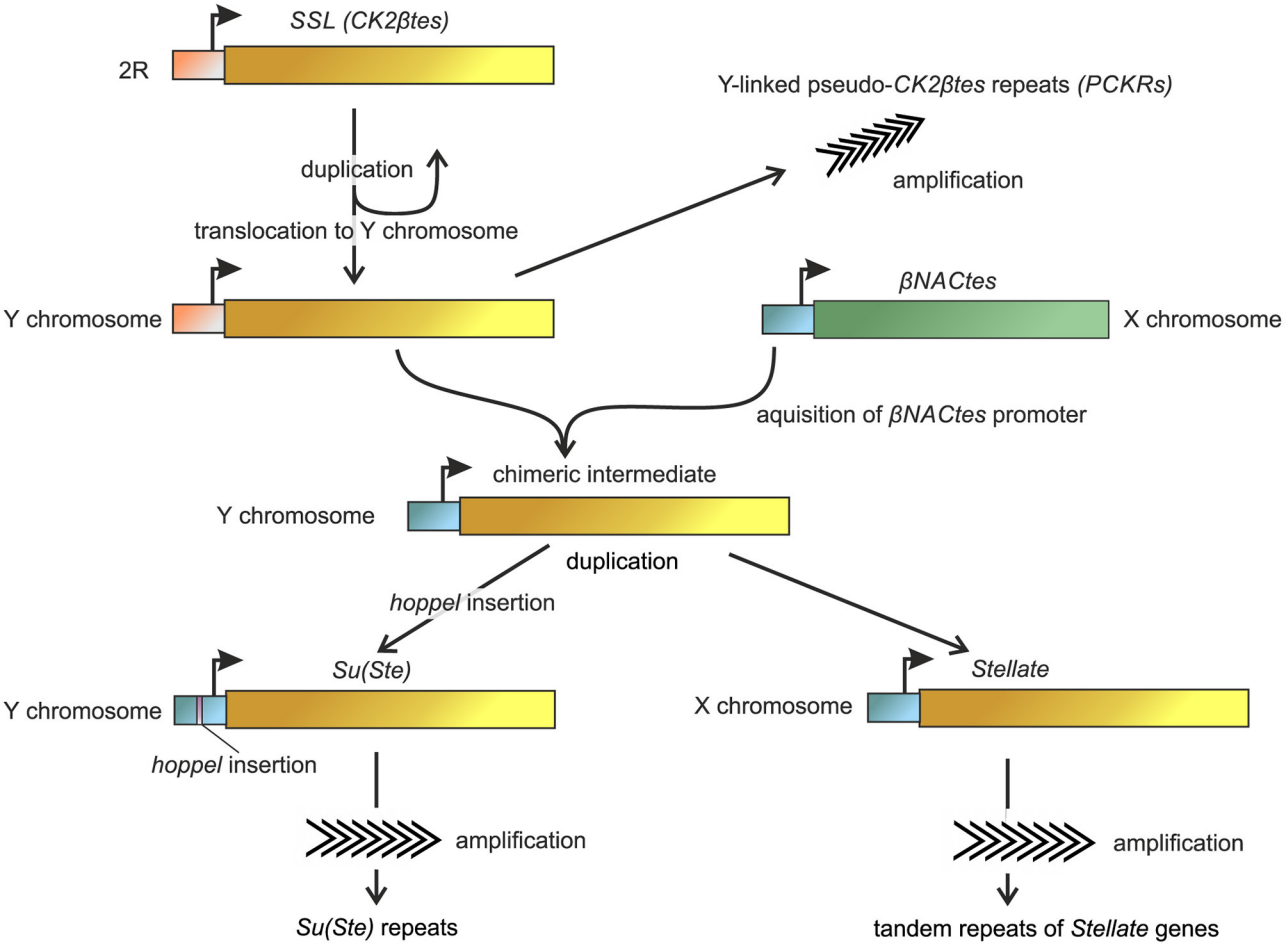
Reconstruction of the basic steps of the origin and evolution of *Ste-Su(Ste)* repeats in the *D. melanogaster* genome. Non-homologous recombination between the promoter of β*NACtes* genes and Y-linked β*CK2tes* copy led to the formation of a chimeric intermediate. Subsequently, the ancestral copies with the acquired promoters were amplified in the sex chromosomes. Insertion of the *hoppel* transposon in the *Su(Ste)* promoter region allowed the acquisition of *Stellate* repressor functions. The homologous sequences are marked by the same color. The model is developed with the usage and modification of ones from Usakin et al. (2005), Chang and Larracuente (2019).

Analysis of published data allows to partially reconstruct the origin and evolution of the *Ste-Su(Ste)* system in the *D. melanogaster* genome (Figure 3). A unique gene mapping to the 60D1-2 euchromatic region of the 2nd chromosome is considered a precursor of the *Ste-Su(Ste)* family (Kalmykova et al., 1997). This gene, called *SSL* (*Su(Ste)*-like) or *CK2*β*tes*, encodes a functional testis-specific β-subunit of the protein kinase CK2 (Kalmykova et al., 1997, 2002). Identical exon-intron structure of the *CK2*β*tes* and *Stellate* genes is observed. The introns of *CK2*β*tes* and *Stellate* are of the same length and are highly homologous. The amino acid sequence of the CK2βtes shows 45% identity to CK2β and 53% identity to Stellate protein. However, the C-terminus of CK2βtes is 40 amino acids longer than that of Stellate. It has been assumed that *CK2*β*tes* gene itself originated from a retroposition of a semi-processed transcript of the canonical β-subunit of protein kinase CK2 (*CK2*β). The retroposition appears to be accompanied by the loss of all introns except the fourth one, which remains conserved for both *CK2*β and *CK2*β*tes*. At the same time, a new intron was acquired in the non-conserved 5′-region of the *CK2*β*tes* ORF. High homology between *CK2*β*tes* and *Stellate*, along with the results of the analysis of the ratio between non-synonymous and synonymous substitutions, suggests that *Ste-Su(Ste)* repeats originated from *CK2*β*tes* via translocation from the autosome to the sex chromosomes and subsequent amplification (Kalmykova et al., 1997).

However, the promoter regions of *CK2*β*tes* gene and *Ste-Su(Ste)* family do not show sequence similarity to each other. BLAST search revealed extremely high homology of the promoter sequences of *Ste-Su(Ste)* repeats with the promoter of an unrelated gene family located as a single cluster in 12DE region of the X chromosome (Figure 3). These genes maintain testes-specific expression and were called β*NACtes* owing to their homology with *bicaudal* gene, which encoding β-subunit of NAC (Nascent polypeptide-Associated Complex) (Usakin et al., 2005). Alignment of the promoters of the *Ste-Su(Ste)* and β*NACtes* families demonstrates a striking similarity of 180 nt nucleotide regions before the transcription start site—up to 95% identity. Taken together with the data about testis-specific expression of a transgene construct containing a 134 bp part of the promoter region of *Stellate* gene (Aravin et al., 2004), these results confirm the defining role of the new promoter region for testis-specific transcription of both gene families, *Ste-Su(Ste)* and β*NACtes*. The common promoter of *Ste-Su(Ste)* and β*NACtes* genes has been functionally mapped and three *cis*-regulatory E-box sites have been determined within this region. Transgene constructs carrying the promoter with the disruption of all three E-boxes do not express the reporter in the testes (Olenkina et al., 2012a,b). E-boxes are known as *cis*-acting elements for the recognition by transcription factors of the basic helix-loop-helix (bHLH) family (Ledent and Vervoort, 2001), and one of them, dUSF, from testis nuclear extract, is able to interact with oligonucleotide fragments of the *Stellate* promoter *in vitro* (Olenkina et al., 2012a). β*NACtes* genes are present in all closely related *Drosophila* species and are considered to be more ancient than the *Ste-Su(Ste)* system (Usakin et al., 2005; Kogan et al., 2012). Thus, a precursor of the *Ste-Su(Ste)* family captured an alien promoter during evolution of *D. melanogaster* to acquire testis-specific expression.

The first step of precursor generation of the *Ste-Su(Ste)* repeats appears to occur through a duplication of *SSL* (*CK2*β*tes*) gene and translocation of the latter to the Y chromosome (Danilevskaya et al., 1991). Indeed, recently a set of 122 pseudogenes that are similar to the initial duplication of *SSL* and carry the ancestral promoter was mapped on the Y chromosome of the new assembly (Chang and Larracuente, 2019). They were designated as pseudo-*CK2*β*tes* repeats on the Y chromosome (*PCKRs*) (Figure 3). *PCKRs* are located proximal to *Su(Ste)* repeats, between *WDY* and *Pp1-Y1* genes (Chang and Larracuente, 2019). A presumed subsequent *Ste-Su(Ste)* evolution step is the creation of a chimeric intermediate, as a result of putative non-homologous recombination between the X-linked β*NACtes* promoter and the Y-linked *SSL* gene. In support of this hypothesis, sequenced scaffold AE003039 has been found earlier in the non-annotated region of the Y chromosome that possesses a pseudogene that could be considered as a chimeric precursor of the *Ste-Su(Ste)* family (Usakin et al., 2005). This pseudogene carries the β*NACtes*-derived promoter fused with a damaged ORF having exon-intron structure which is identical to *Ste-Su(Ste)-SSL*. The 5′-region upstream the transcription start site of this pseudogene contains a fragment of about 200 nt with high homology to the sequence adjacent to the promoter of the β*NACtes3* gene and other sequences specific for the β*NACtes* cluster (Usakin et al., 2005), implying that a recombination event may occur between the X and Y chromosomes. Following the initial duplication of *SSL* in the Y chromosome, the ancestral copies with the acquired testes-specific promoters were amplified independently in the sex chromosomes with the possible participation of the *Helitron* family transposable elements (Kogan et al., 2012), which led to the formation of both euchromatic and heterochromatic clusters of *Stellate* genes on the X chromosome and *Su(Ste)* repeats on the Y. The X-linked *Stellate* genes and Y-linked *Su(Ste)* copies were amplified independently. The insertion of the *hoppel* transposon in the *Su(Ste)* promoter region is considered as a key event responsible for the acquisition of repressor functions by *Su(Ste)* repeats. This insertion defined the antisense transcription of *Su(Ste)* repeats and subsequent generation of *Su(Ste)* piRNAs (Aravin et al., 2001, 2004). Note that the promoter region of heterochromatic *Stellate* genes contains no traces of *hoppel* insertion and is closest to the parental β*NACtes* promoter. However, euchromatic *Stellate* genes carry a 16 bp deletion in the corresponding region of the promoter, which could be a sign of the insertion and subsequent imperfect excision of *hoppel* (Olenkina et al., 2012a). This data argues for the hypothesis according to which ancestral copies of euchromatic and heterochromatic *Stellate* clusters translocate on the X chromosome independently and at different stages of the evolutionary history of the *Ste-Su(Ste)* family. Phylogenetic analysis provided recently (Chang and Larracuente, 2019) supports that euchromatic and heterochromatic *Stellate* clusters are amplified independently of each other. Both euchromatic and heterochromatic *Stellate* repeats are fixed in the *D. melanogaster* genome and contain intact ORFs (Tulin et al., 1997; Kogan et al., 2000). Polymorphism in their coding regions is mainly determined by synonymous substitutions. Apparently, translational selection supports high homogeneity of *Stellate* genes. Intra-locus divergence between adjacent heterochromatic *Stellate* genes is nearby 0.1–0.2% (Tulin et al., 1997), and divergence between randomly selected *Stellate* pairs does not exceed 2.5% (McKee and Satter, 1996).

It should be noted that the *Ste-Su(Ste)* genetic system is found only in *D. melanogaster*. The genomes of three closely related *Drosophila* species have diverged from *D. melanogaster*. Analysis of recent genome assemblies of the *simulans* clade species reveals that *pseudo-*β*CK2tes repeat* (*PCKR*) duplications are present in the Y chromosome of all three sibling species, *D. simulans, D. mauritiana*, and *D. sechellia*, in the range from 22 to 117 copies (Chakraborty et al., 2020). These findings allow suggesting that the first stages of the *Ste-Su(Ste)* system formation occurred before the splitting of *D. melanogaster* from the common precursor, but subsequent dynamics of the evolution process led to species diversification.

## Properties of Stellate Proteins

The derepression of *Stellate* genes causes the emergence of protein crystals both in the cytoplasm and nuclei of spermatocytes of *cry*^1^ males. Stellate appears to be the predominant or the exclusive component of these crystals (Bozzetti et al., 1995, Egorova et al., 2009). The needle-like shape of crystalline aggregates indicates their generation in a head-to-tail manner (Figures 2A,D). Mass-spectrometry analysis points to the presence in their content of protein products of both euchromatic and heterochromatic *Stellate* clusters (Olenkina et al., 2012b). Since Stellate protein possesses 38% homology with the canonical regulatory β-subunit of the protein kinase CK2 (CK2β) (Livak, 1990; Bozzetti et al., 1995), it is suggested that Stellate is able to interact with the catalytic α-subunit of CK2 (CK2α) in certain conditions. Protein kinase CK2 (also known as casein kinase 2) is a multi-functional ubiquitous heterotetrameric α2β2 complex, which takes part in numerous signaling cascades, cell differentiation, proliferation and surviving (Pinna, 2002; Bibby and Litchfield, 2005; Bandyopadhyay et al., 2016). CK2 phosphorylates protein substrates at serine or threonine residues embedded in an S/TXXD/E motif, where X could be any amino acid residue except the basic ones (Allende and Allende, 1995). Among the established CK2 targets in *D. melanogaster* there are nuclear proteins such as HP1 (Heterochromatin Protein 1) (Zhao and Eissenberg, 1999), topoisomerase II (Ackerman et al., 1985, 1988), Mi-2 (Bouazoune and Brehm, 2005), transcription factor GAGA (Bonet et al., 2005), and other proteins with a predominance of transcription factors (Bandyopadhyay et al., 2016). The regulatory subunits CK2β modulate the substrate specificity of CK2α and its phosphotransferase activity and also ensure the stability of the holoenzyme (Bibby and Litchfield, 2005).

Bozzetti and colleagues showed that recombinant Stellate protein was able to interact *in vitro* with recombinant CK2α, and its 17-fold excess somewhat stimulated the basal activity of CK2α (Bozzetti et al., 1995). However, the functional interaction of Stellate present in the form of insoluble aggregates in spermatocytes with CK2α seems to be problematic and doubtful. Accordingly, the two-hybrid assay did not detect any interaction of Stellate with CK2α, in contrast to canonical CK2β subunits of *D. melanogaster*, testis-specific subunits CK2β′, and CK2βtes (SSL) (Karandikar et al., 2003). Stellate protein lacks the conserved C-terminal domain with the aid of which CK2β interacts with CK2α. Unlike the other β-subunits of *Drosophila*, Stellate was not able to compensate for the absence of CK2β and did not rescue the defects of ionic homeostasis when it was expressed in *Saccharomyces cerevisiae* strains with deletion of yeast genes encoding their own β-subunits (Karandikar et al., 2003). However, biochemical and immunofluorescence studies have shown that hyperexpression of Stellate protein causes the accumulation Stellate crystalline aggregates mainly in the cytoplasm of premeiotic spermatocytes, whereas soluble Stellate protein is clearly detected in the spermatocyte nuclei of the *cry*^1^ testes (Egorova et al., 2009) (Figures 2A,C). The soluble Stellate appears in the spermatocytes in the mid-G2 phase and persists throughout meiotic interphase. It is coimmunoprecipitated with CK2α, despite the lack of canonical CK2β C-terminal domain (Egorova et al., 2009), which indicates possible formation of heterotetrameric holoenzyme CK2 via alternative contacts. The biological significance of this interaction is not clear. It is possible that at some stage of *D. melanogaster* evolution the appearance of *Stellate* gene encoding the alternative testis-specific β-subunit of CK2 was supported by positive selection, but later its functions turned out to be dispensable or harmful.

One more surprising circumstance is in agreement with this assumption. Immunochemical and mass-spectrometry data confirmed that both forms of Stellate proteins, soluble and crystalline, undergo trimethylation of lysine residue K92. Endogenous Stellate proteins are recognized with a high affinity by antibodies against histone H3 trimethylated at K9 lysine residue (Egorova et al., 2009). Thus, the methylated site of Stellate proteins mimics the epigenetic modification of histone H3, H3K9me3, that is a hallmark of transcriptionally repressed heterochromatin (Kouzarides, 2007; Allis and Jenuwein, 2016; Ninova et al., 2019). Three histone methyltransferase, Su(var)3-9, dG9a, and dSETDB1, are involved in the H3K9 methylation in *Drosophila* (Tachibana et al., 2001; Schotta et al., 2002; Schultz et al., 2002). While Stellate does not exhibit similarity to the corresponding histone H3 sequence (–TAR**K9**ST–), the methylated site of Stellate, –MHR**K92**YL/M–, contains an –RK– dipeptide motif that has been shown to be the principal determinant for lysine methylation provided by methyltransferase G9a (Rathert et al., 2008). The site for lysine methylation has emerged and has been fixed only in *Stellate* sequence because sequences of other *Drosophila CK2*β subunits, including *CK2*β*tes*, do not contain it (Egorova et al., 2009). The functional significance of the trimethylation of Stellate is still obscure. Proteins of the highly conserved Heterochromatin Protein 1 family are known as H3K9me3 readers aided by their N-terminal chromo domain (Vermaak and Malik, 2009; Ninova et al., 2019). The H3K9me3 modification is recognized by HP1 that is essential for the formation of heterochromatin genomic regions (Fischle et al., 2005; Kouzarides, 2007; Vermaak and Malik, 2009). Both hypo- and hyper-expression of HP1 lead to disturbances in the compaction of pericentromeric heterochromatin and abnormalities in centromeric cohesion and segregation of chromosomes (Inoue et al., 2008; Vermaak and Malik, 2009). Besides the K92me3 site mimicking the H3K9me3 mark, Stellate also possesses two -P(L)XVXL- motifs, known as potential sites for interaction with the chromo shadow domain of HP1 (Lechner et al., 2005). Taking into account the above-mentioned observations, it can be proposed that thrimethylated Stellate is able to interact with HP1 or other chromo domain-containing proteins in premeiotic spermatocytes. However, the functional significance of these possible interactions is unexplored to date.

## *Stellate* Genes as a Major Target of Pirna Silencing in the Testes of *D. Melanogaster*

At the beginning of the XXI century, in 2001, a new class of small non-coding RNAs, piRNAs, 23–30 nt in length, associated with silencing of *Stellate* genes has been discovered in the testes of *Drosophila melanogaster* by Aravin and colleagues (Aravin et al., 2001). The piRNA pathway is conserved in a wide range of animals from fungi to mammals (Aravin et al., 2007; Malone et al., 2009; Gainetdinov et al., 2018). The piRNA pathway provides both innate and adaptive immune defense against the activity of transposable elements protecting genome integrity in germinal tissues. It also contributes to the maintenance of germline stem cells, ensures expression regulation of protein-coding genes, functions in the establishment of embryonic patterning (in *Drosophila*), and provides trans-generational epigenetic inheritance (Malone et al., 2009; Le Thomas et al., 2014; Rojas-Ríos et al., 2017; Rojas-Ríos and Simonelig, 2018; Ozata et al., 2019). The piRNAs mainly function in the gonads and are characterized by extreme diversity existing as millions of unique piRNA sequences (Huang et al., 2017), unlike other main classes of small non-coding RNAs, siRNAs and microRNAs. RNA-induced silencing complexes (RISCs) operate in all pathways with the participation of small RNAs, and ARGONAUTE proteins are the key players of different RISCs (Czech and Hannon, 2016; Huang et al., 2017). piRNAs load to PIWI subfamily of ARGONAUTE family proteins: Piwi, Aubergine (Aub) and ARGONAUTE 3 (AGO3) in *Drosophila* (Aravin et al., 2001; Vagin et al., 2006; Li et al., 2009; Malone et al., 2009). AGO3 and Aub are expressed only in the germline, while Piwi is also found in somatic cells of gonads. Complementary base pairing with a small RNA guides the piRISC to a specific RNA target, generally providing silencing via its direct cleavage or translational repression. Mature piRISC complexes have a different destiny depending on the protein component. Piwi loaded by piRNA enters the nucleus, where it participates in co-transcriptional silencing, whereas Aub and AGO3 reside in cytoplasmic granules and function in post-transcriptional silencing.

**Figure 4:**
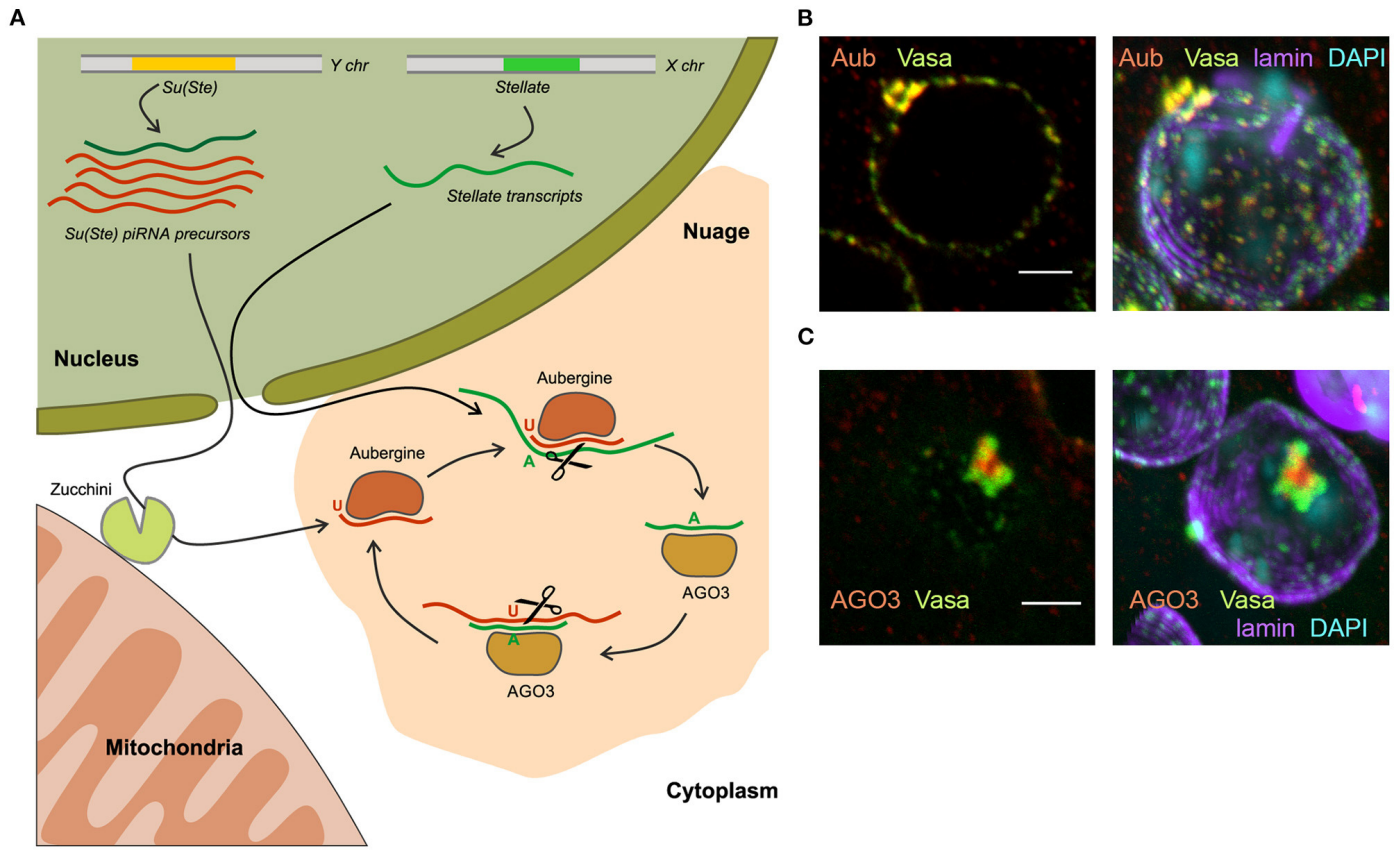
The piRNA biogenesis in the *nuage* granules of *Drosophila* spermatocytes. **(A)** Long transcripts of *Su(Ste)* piRNA precursors are exported from the nucleus and are presumably cleaved by endonuclease Zucchini located on the outer membrane of mitochondria, forming the 5′-end of the future piRNA. The cleaved transcript is loaded into Aubergine, and then trimmed from the 3′-end by an unknown trimmer nuclease. Aubergine loaded with guide antisense piRNAs recognizes and cleaves the complementary *Stellate* mRNAs producing the 5′-end of a new sense piRNA. The new piRNA is loaded into AGO3 and in turn performs cleavage of the complementary *Su(Ste)* transcript. This step generates a new antisense piRNA that is identical or very similar to the initiating piRNA (ping-pong cycle). **(B)** Vasa and Aubergine are colocalized in the periphery lobes of the piNG-body. **(C)** AGO3 is located in the central lobe of the piNG-body and does not colocalized with Vasa. **(B,C)** Confocal slices (left) and 3D images (right) of premeiotic spermatocytes in testis preparations are reproduced from Figure 3 in Kibanov et al. (2011) by permission of MBoC. Under the License and Publishing Agreement, authors grant to the general public, the non-exclusive right to copy, distribute, or display the manuscript subject to the terms of the Creative Commons-Non-commercial-Share Alike 3.0 Unported license (http://creativecommons.org/licenses/by-nc-sa/3.0). Scale bars are 3 μm.

Early classification of piRNAs based on the peculiarities of their biogenesis divided piRNAs into two main groups: primary and secondary piRNAs. The main sources of primary piRNAs are long transcripts originating from piRNA clusters, specific genome loci residing mainly in heterochromatin and generally representing transposon “graveyards” (Brennecke et al., 2007; Czech and Hannon, 2016; Huang et al., 2017). In germ cells of *Drosophila* gonads primary or maternally inherited piRNAs initiate the formation of secondary piRNAs. The cyclic mechanism called “ping-pong” provides amplification of the piRNA pool for the rigorous repression of selfish element activity. Ping-pong was discovered in *Drosophila* ovarian nurse cells (Brennecke et al., 2007) and was subsequently defined as the conserved property of the piRNA pathway (Aravin et al., 2007; Gainetdinov et al., 2018). Piwi- and Aub-associated piRNAs are found to be enriched with uridine at the 1st position from the 5′-end. AGO3-related piRNAs are enriched with adenosine at the 10th position and do not have a clear preference for the 1st position. Consistent with the initially proposed model, Aub complexed with a primary antisense piRNA, derived from a piRNA cluster transcript, recognizes and trims the transcript of a transposable element, creating the 5′-end of a secondary sense piRNA. All PIWI proteins cleave their target transcripts between nucleotides 10 and 11 of the paired piRNA guide. Thus, the first 10 nucleotides of a secondary piRNA possess complementarity to the first 10 nucleotides of a primary piRNA directing the cleavage act. This secondary piRNA precursor is loaded into AGO3 and matures via trimming at the 3′-end. The sense piRNA-AGO3 complexes in turn, recognize and process a long transcript of a piRNA cluster to form a new antisense piRNA with the sequences that are identical or very similar to the original primary one. This process is repeated cyclically (Figure 4A). Ping-pong processing increases the piRNA pool quantitatively, but it does not create new piRNA sequences. The maturation of piRNA intermediates, associated with Aub or Piwi, can also take place through the production of phased piRNAs from adjacent regions of the transcript (Han et al., 2015; Mohn et al., 2015; Senti et al., 2015; Wang et al., 2015). It can be considered as a mechanism of adaptation to the targets through diversification of piRNA sequences. Thus, at least in the germline, the separation of primary and secondary piRNA biogenesis is rather provisional, since both variants of processing are more closely related than previously thought (Czech and Hannon, 2016; Huang et al., 2017; Gainetdinov et al., 2018).

Cytoplasmic electron-dense perinuclear granules called *nuage* are considered to be centers of piRNA biogenesis and piRNA-mediated post-transcriptional silencing in the germline of *Drosophila*. To date, the organization and functions of the *nuage* are described in some details (Findley et al., 2003; Snee and Macdonald, 2004; Lim and Kai, 2007; Lim et al., 2009; Patil and Kai, 2010; Kibanov et al., 2011; Nosov et al., 2014; Webster et al., 2015). Among the known components that are concentrated in the *nuage*, three classes of proteins prevail: PIWI proteins (Aub and AGO3), RNA helicases, and Tudor-domain-containing proteins. The *nuage* contains RNA as an essential component. A growing body of evidence suggests that *nuage* formation is based on the principles of liquid–liquid phase separation (Brangwynne et al., 2009; Nott et al., 2015). A number of proteins are necessary for piRNA-mediated repression of *Stellates* in the testes and retrotransposons in the ovarian germline are located in or associated with the *nuage*: Aub, AGO3, Vasa, Spindle-E, Armitage, Tudor, Tejas, Krimper, Maelstrom, Squash, Qin/Kumo, Zhuccini, etc. (Aravin et al., 2004; Vagin et al., 2006; Lim and Kai, 2007; Pane et al., 2007; Malone et al., 2009; Patil and Kai, 2010; Kibanov et al., 2011; Zhang et al., 2011; Anand and Kai, 2012). Vasa and Spindle-E are ATP-dependent RNA helicases. Vasa takes part in the different stages of the piRNA silencing, it presumably mediates nuclear export of piRNA precursors (Zhang et al., 2012) and has basic architectural functions in *nuage* formation, being the top component of the *nuage* hierarchy. *vasa* mutations lead to a loss of *nuage* granules, mislocalization of Aub, AGO3 and other components in the cytoplasm, and to the disruption of piRNA silencing both in the ovaries and testes (Lim and Kai, 2007; Li et al., 2009; Malone et al., 2009; Patil and Kai, 2010; Kibanov et al., 2011). Tudor, Spindle-E, Qin/Kumo, Tejas and Krimper proteins possess the TUDOR-domains, which recognizes post-translationally methylated lysine residues in PIWI proteins, which are essential for the *nuage* assembly (Kirino et al., 2009; Nishida et al., 2009; Kibanov et al., 2011).

In *Drosophila* spermatocytes, among small *nuage* granules, a large granule, the piNG-body was uncovered, that was more than 50 times larger than the smaller ones (Kibanov et al., 2011). The piNG-body is present in primary spermatocytes as one giant *nuage* granule per cell. piNG-bodies move around the outer surface of the nuclei (Nosov et al., 2014) and persist in spermatocytes throughout the entire meiotic interphase. The large size of the piNG-body allowed to determine a specific pattern of piRNA pathway component distribution: Spindle-E, Aub, and Tudor are colocalized with Vasa in the periphery lobes of the piNG-body, while AGO3 is found only in the central lobe (Kibanov et al., 2011, 2013; Ryazansky et al., 2016) (Figures 4B,C). These results are consistent with data indicating different subcellular localization of Aub and AGO3 and independent mechanisms of their recruitment to the *nuage* in the ovaries (Webster et al., 2015). With the aid of fluorescence recovery after photobleaching (FRAP) experiments, the existence of a dynamic exchange of GFP-tagged Vasa between the piNG-body and the cytoplasm in spermatocytes under normal conditions was shown (Nosov et al., 2014). A similar exchange of Vasa, Aub, AGO3, Tudor, Tejas, and Spindle-E molecules was observed between the *nuage* and their cytoplasmic pool in the ovaries (Snee and Macdonald, 2004; Xiol et al., 2014; Webster et al., 2015). Only 71% recovery after photobleaching of the Vasa-GFP signal in the piNG-bodies indicates the existence of at least 29% stationary fraction that is not involved in the rapid exchange with the cytosolic mobile fraction (Nosov et al., 2014). Liquid–liquid phase separation is proposed to slow this exchange down owing to the higher viscosity of *nuage* granules supported by specific protein-protein interactions (Ozata et al., 2019).

Historically, the first example of piRNA-mediated gene repression is *Stellate* gene silencing in the *Drosophila* testes with the aid of piRNAs derived from Y-linked *Su(Ste)* repeats (Aravin et al., 2001). To date, the mechanism of *Sellate* repression has been clarified in some details. *Su(Ste)* represents the major testis piRNA cluster producing about 43% of total piRNAs (Kotov et al., 2019; Chen et al., 2020) unlike the situation in the ovarian germline, where *42AB* in pericentromeric region of the 2nd chromosome is found to be the most active piRNA cluster (Malone et al., 2009). Chronologically, antisense *Su(Ste)* transcripts appear in the nuclei of early spermatocytes before the sense ones. Antisense transcription is initiated from several sites within the *hoppel* transposon inserted in the *Su(Ste)* promoter (Aravin et al., 2001, Figure 1). In the nuclei of mature primary spermatocytes sense and antisense *Su(Ste)* transcripts are found to be colocalized (Aravin et al., 2004). However, antisense *Su(Ste)* transcripts are much more abundant (more than 20-fold) than sense ones and provide a great amount of piRNAs with high complementarity to *Stellate* transcripts (Kotov et al., 2019). *Stellate* gene transcription starts in early spermatocytes. Deletion of the bulk part of *Su(Ste)* caused increased accumulation of spliced, but not non-spliced *Stellate* transcripts in the nuclei and cytoplasm of spermatocytes (Kotelnikov et al., 2009), indicating the post-transcriptional mode of *Stellate* silencing. Analysis of *Su(Ste)-Stellate* piRNAs reveals strong U1 (78%) and A10 (54%) nucleotide biases for antisense and sense piRNAs, respectively. This points to the existence of piRNA pairs with a 10-nt overlap generated through the ping-pong mechanism (Ryazansky et al., 2016; Kotov et al., 2019, Figure 4A). Expression and silencing of *Stellate* genes coincide with the organization of the piNG-bodies, that contain the components of the piRNA machinery in a high concentration. Mutations of *vasa, aub* and *ago3* cause the destruction of the piNG-bodies that results in *Stellate* derepression. Note that the *ago3* mutation does not lead to the disappearance of the small nuage granules, indicating that a high concentration and compartmentalization of the piRNA pathway components achieved in the piNG-body are essential for successful silencing (Kibanov et al., 2011).

The piRNA pathway has evolved to keep a high level of sequence complementarity between a piRNA and its target in the germline (Kotov et al., 2019; Chen et al., 2020). Expression of *Stellate* genes is perfectly repressed by *Su(Ste)* piRNAs in the testes of wild-type males. Other gene encoding testis-specific β-subunit of CK2, *CK2*β*tes*, possesses a significant homology with *Su(Ste)* and *Stellates*, about 70% identity of nucleotide sequence. However, *CK2*β*tes* is expressed in the male germline at a high level (Kalmykova et al., 2002, 2005) and does not undergo targeting by *Su(Ste)* piRNAs. The second major piRNA cluster in the testes, *AT-chX*, resides in the pericentromeric region of the X chromosome and also contains internal tandem repeats (Kotov et al., 2019; Chen et al., 2020). This cluster produces abundant and diverse piRNAs with 76% homology to *vasa* transcripts mainly in antisense orientation. However, in contrast to the previously published data (Nishida et al., 2007) we found that sequence similarity between *AT-chX*-derived piRNAs and *vasa* transcripts is not enough for their repression in the testes and ovaries of *D. melanogaster* (Kotov et al., 2019). On the whole, these data suggest that effective piRNA silencing at least in premeiotic germ cells of *Drosophila* testes requires a high level of sequence complementarity between piRNAs and their targets to prevent harmful off-target effects.

## Contribution of the *Ste-Su(Ste)* System to Hybrid Sterility and Reproductive Isolation Between *D. MELANOGASTER* and Closely Related Species

Despite the deep insight into the evolution and regulation of *Stellate* genes expression, functions and biological significance of the *Ste-Su(Ste)* system have been obscure for a long time. According to one early hypothesis, the *Ste-Su(Ste)* system exists as a parasitic self-maintaining genetic system that only mimics functions crucial for meiosis (Bozzetti et al., 1995). According to another assumptions, the *Ste-Su(Ste*) system is similar to toxin-antitoxin systems, which are widely found in prokaryotes (Aravin, 2020). However, the most common fate for insignificant duplicated genes is their rapid degeneration and loss of function (Conant and Wolfe, 2008). The energy-consuming maintenance of multiple highly homogenous copies of *Stellate* genes in the genome and their permanent silencing indicate the existence of selective forces that prevent the loss of long-lived *Stellate* genes. Thus, the *Ste-Su(Ste)* system is fixed in the *D. melanogaster* genome and maintained under positive selection. *Stellate* genes are normally completely repressed in the male germline by piRNAs generated from Y-linked *Su(Ste)* repeats. A deletion of the *cry*^1^ locus or a loss of the entire Y chromosome leads to *Stellate* derepression in spermatocytes and to subsequent meiotic disorders causing complete or partial male infertility. Tandem *Stellate* genes are only found in the *D. melanogaster* genome, but not in other *Drosophila* species, as mentioned above. We have hypothesized that *Stellate* genes may function in the reproductive isolation of *D. melanogaster* from closely related species lacking *Su(Ste)* repeats in the Y chromosomes.

Highly homogenous *AT-chX* repeats are also fixed in *D. melanogaster* and not found in other *Drosophila* species. Strikingly, we revealed that *vasa* sequences from closely related species, *D. simulans, D. sechellia* and *D. mauritiana*, that have diverged from an ancestor common with *D. melanogaster* 2.0–5.4 million years ago (Russo et al., 1995; Tamura et al., 2004), maintain more than 90% complementarity with piRNAs from *AT-chX* repeats of *D. melanogaster* compared with only 76% for *vasa* of *D. melanogaster* itself (Kotov et al., 2019). In line with this observation, we have proposed that *AT-chX* piRNAs could repress alien *vasa* transcripts in the gonads of interspecies hybrids.

**Figure 5:**
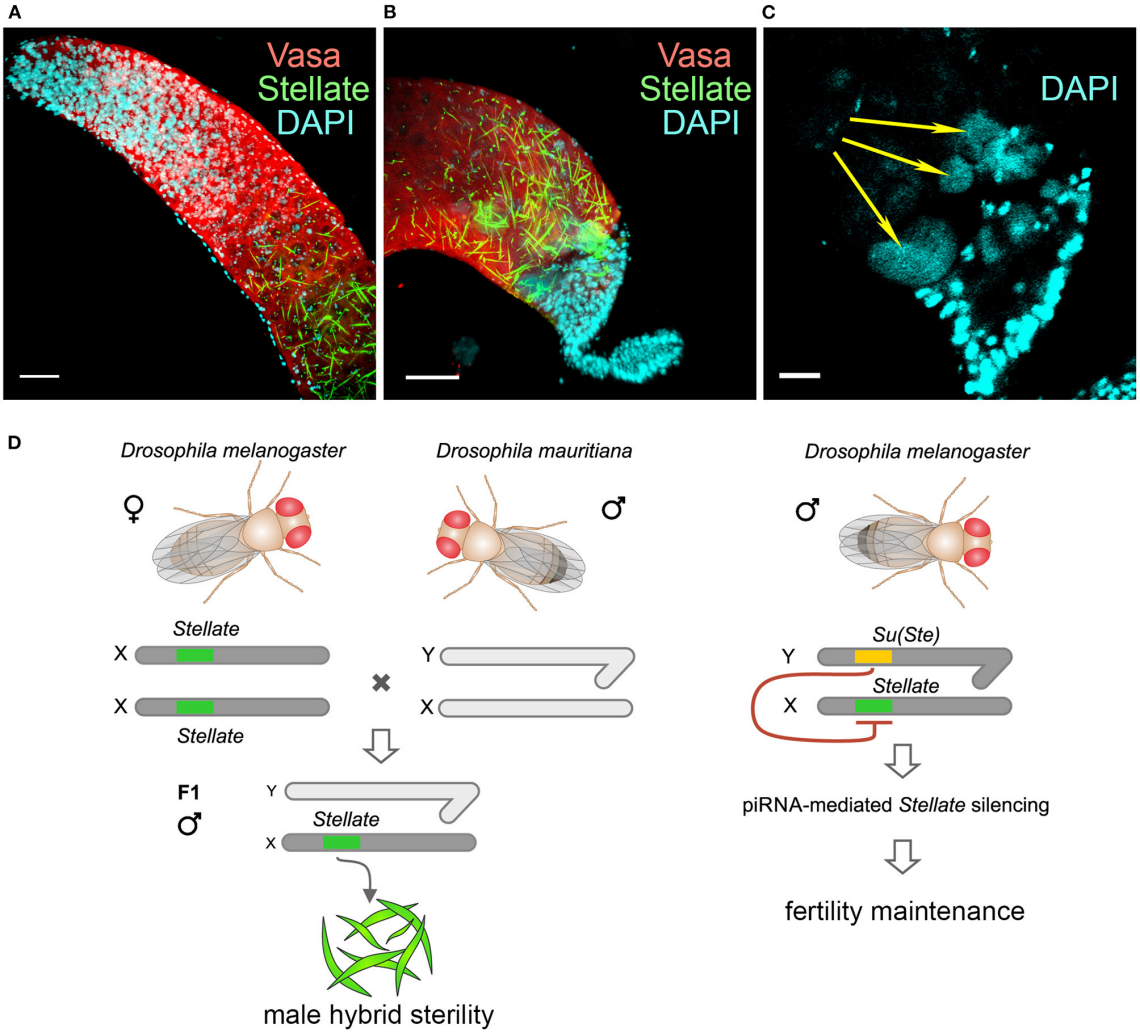
Derepression of *Stellate* genes in the testes of interspecies hybrids *D. melanogaster* with *D. mauritiana*. **(A–C)** Hybrid testes were stained with anti-Vasa (red) and anti-Stellate (green) antibodies, chromatin was stained with DAPI (cyan). 3D images are present for apical **(A)** and distal **(B)** testes ends; an internal confocal slice is present for the distal testis end stained with DAPI **(C)**. Scale bars are 30 μm for **(A)** and **(B)**, 15 μm for C. *Stellate* genes are derepressed in spermatocytes of hybrid testes and generate abundant crystals **(A,B)**. Spermatids are absent at the distal end of hybrid testes **(B)**. Giant conglomerations of decondensed chromatin (yellow arrows) are found at the distal ends of hybrid testis **(C)** indicating a severe meiotic catastrophe. **(D)** Derepession of *Stellate* genes in the testes of hybrids causes meiotic failures and male hybrid sterility. **(A–D)** Images are reproduced from Figure 6B in Kotov et al. (2019), and the scheme is adapted from Figure 7B in Kotov et al. (2019) by permission of Oxford University Press (http://global.oup.com/academic) without the need to obtain written permission from OUP and payment as authors of this publication.

The studying of viable interspecific hybrids is a way to identify hybrid incompatibility factors that cause reproductive isolation between species. It has been shown earlier that the *Hmr* mutation significantly rescues survival of F1 male hybrid progeny of crosses between *D. melanogaster* females and males of the *simulans* clade (Barbash et al., 2000; Barbash and Ashburner, 2003). We raised hybrid males by crossing *Hmr*^1^ *D. melanoga*ster females and *D. mauritiana* males to test our hypotheses (Kotov et al., 2019). The hybrid males inherit the maternal X chromosome carrying *Stellate* genes and the *AT-chX* piRNA cluster, and the paternal Y chromosome that does not contain *Su(Ste)* repeats. We uncovered that the piRNA biogenesis was not disrupted in the testes of hybrids, because the analysis of small RNAs reveals the presence of 23–30 nt piRNAs mapped to various transposable elements (Kotov et al., 2019). These piRNAs exhibit U1-bias for antisense piRNAs, whereas the sense ones possess A10-bias, indicating that the piRNA pathway is still functional. We have observed a strong silencing of *D. mauritiana vasa* by trans-acting piRNAs from the *AT-chX* cluster in hybrid testes, whereas *vasa* of *D. melanogaster* does not undergo repression. The majority of hybrid testes has reduced size and contains just a few or no germ cells. We assumed that selective repression of one allele of the *vasa* gene in diploid hybrids might cause haploinsufficiency of *vasa* functions and disruption of germ cell development at early stages, appearing to contribute to hybrid male sterility. However, the molecular basis of hybrid dysfunction is not characterized yet in this case. For the analysis of *Stellate* gene regulation we selected only perfectly developed hybrid testes with maximal germ cell content (Kotov et al., 2019). These wild-type size testes are filled a large number of germ cells at different stages of spermatogenesis. Expectedly, *Su(Ste)* piRNAs are not generated in the testes of the hybrids. Immunostaining of the testis preparations reveals the presence of abundant Stellate aggregates in spermatocytes of the hybrid testes (Figures 5A,B), but not in the testes of parent flies. Spermatocytes in the hybrid testes do not progress through meiosis, haploid spermatids are absent at the distal end of the testes and the seminal vesicles do not contain mature sperm. Giant conglomerations of decondensed chromatin at the distal end of these testes indicate a severe meiotic catastrophe (Kotov et al., 2019, Figure 5C). Thus, strong *Stellate* derepression in the testes of hybrid males definitely leads to their infertility.

## Conclusions and Perspectives

One of main condition of species splitting from a common precursor lineage is the prevention of gene flow between diverging populations. Thus, reproductive isolation caused by hybrid lethality or sterility is a necessary condition for speciation (Orr, 2005; Castillo and Barbash, 2017). Postzygotic reproductive isolation emerges as a consequence of gradual accumulation of genetic differences between isolated populations of the ancient precursor. These differences, emerging due to selection or genetic drift, lead to lower fitness or sterility of hybrid progeny. The well-known Dobzhansky–Muller model suggests that a common cause of genetic incompatibility between closely related species is the divergence among alleles at two or more loci (Dobzhansky, 1936; Muller, 1940; Muller and Pontecorvo, 1942). We did not identify new Dobzhansky–Muller gene pairs in parental genomes. However, we showed that abnormal regulatory divergence triggered by the piRNA pathway provides two opposite harmful effects in the testes of interspecies hybrids: loss of *Su(Ste)* piRNAs leads to derepression of *Stellate* genes causing defects in male meiosis (Figure 5D), whereas silencing of *D. mauritiana vasa* by *AT-chX* piRNAs originating from the *D. melanogaster* genome appears to contribute to defects of early spermatogenesis stages (Kotov et al., 2019). In accordance with these findings a recently published study leads to the speculation that rapid intrinsic divergence of human pachytene piRNA loci among placental mammals also may be an undescribed driver of reproductive isolation (Özata et al., 2020). It would be attractive to test in future the derepression of *Stellate* genes in the interspecific hybrids of *D. melanogaster* with *D. simulans* or *D. sechellia* as well. However, to date our attempts to cross *D. melanogaster* females with males of these species have resulted in hybrid males with severely reduced testes that did not contain germ cells. This indicates a failure in germ cell maintenance at earlier stages of testis development. It can be proposed that the repression of one of the *vasa* alleles affects this phenotype. But this hypothesis requires a further investigation that is strongly hampered owing to a loss of germ cells. Potential advances in this field can be provided by studies of the ovaries of hybrid females, since the *AT-chX* piRNA cluster is shown to be active in the ovaries of *D. melanogaster* and produce abundant piRNAs (Chen et al., 2020). The generation and usage of female hybrid offspring with developed ovaries may allow to study the impact of *vasa* misregulation on the maintenance of early germ cells and fertility in the hybrid genomic background.

The studies of hybrid male sterility in *Drosophila* allowed to uncover that reproductive isolation can occur due to a small number of genes misexpressed in hybrids (Michalak and Noor, 2003; Haerty and Singh, 2006; Moehring et al., 2007; Wei et al., 2014). The acquisition of the *Ste-Su(Ste)* system by a part of the ancient fly population appears to be one of the causative factors of hybrid sterility in crosses of female flies with males that do not carry Y-linked *Su(Ste)* repeats. In our experiments, we reproduced this putative scenario by directly demonstrating *Stellate* derepression in the background of interspecies hybrids between *D. melanogaster* and *D. mauritiana* (Figure 5). The results embrace our hypothesis about the contribution of the *Ste-Su(Ste)* system and its epigenetic regulation by the piRNA pathway in reproductive isolation of the *D. melanogaster* lineage. By studying the molecular and genetic events that lead to the emergence of reproductive isolation between lineages originating from a single ancestral population, we have pieced together some of the processes that cause *D. melanogaster* divergence from closely related species to improve our understanding of the process of speciation.

## Author Contributions

VA and LO prepared the initial version of the manuscript and created the figures. AK, SB, AS, and AA rigorously revised and improved the manuscript. VA, LO, AK, and AA polished the final version of the manuscript. All authors provided intellectual contribution, edited, and approved the manuscript for publication in its present version.

## Conflict of Interest

The authors declare that the research was conducted in the absence of any commercial or financial relationships that could be construed as a potential conflict of interest.

## References

[B1] Ackerman P. Glover C. V. Osheroff N. (1985). Phosphorylation of DNA topoisomerase II by casein kinase II: modulation of eukaryotic topoisomerase II activity *in vitro*. Proc. Natl. Acad. Sci. U. S. A. 82, 3164–3168. 10.1073/pnas.82.10.3164 2987912 PMC397735

[B2] Ackerman P. Glover C. V. Osheroff N. (1988). Phosphorylation of DNA topoisomerase II *in vivo* and in total homogenates of *Drosophila* Kc cells. The role of casein kinase II. J. Biol. Chem. 263, 12653–12660. 2842338

[B3] Allende J. E. Allende C. C. (1995). Protein kinases. 4. Protein kinase CK2: an enzyme with multiple substrates and a puzzling regulation. FASEB J. 9, 313–323. 10.1096/fasebj.9.5.7896000 7896000

[B4] Allis C. D. Jenuwein T. (2016). The molecular hallmarks of epigenetic control. Nat. Rev. Genet. 17, 487–500. 10.1038/nrg.2016.59 27346641

[B5] Anand A. Kai T. (2012). The tudor domain protein kumo is required to assemble the *nuage* and to generate germline piRNAs in *Drosophila*. EMBO J. 31, 870–882. 10.1038/emboj.2011.449 22157814 PMC3280549

[B6] Aravin A. A. (2020). Pachytene piRNAs as beneficial regulators or a defense system gone rogue. Nat. Genet. 52, 644–645. 10.1038/s41588-020-0656-8 32601474

[B7] Aravin A. A. Klenov M. S. Vagin V. V. Bantignies F. Cavalli G. Gvozdev V. A. (2004). Dissection of a natural RNA silencing process in the *Drosophila melanogaster* germ line. Mol. Cell. Biol. 24, 6742–6750. 10.1128/MCB.24.15.6742-6750.2004 15254241 PMC444866

[B8] Aravin A. A. Naumova N. M. Tulin A. V. Vagin V. V. Rozovsky Y. M. Gvozdev V. A. (2001). Double-stranded RNA-mediated silencing of genomic tandem repeats and transposable elements in the D. melanogaster germline. Curr. Biol. 11, 1017–1027. 10.1016/S0960-9822(01)00299-8 11470406

[B9] Aravin A. A. Sachidanandam R. Girard A. Fejes-Toth K. Hannon G. J. (2007). Developmentally regulated piRNA clusters implicate MILI in transposon control. Science 316, 744–747. 10.1126/science.1142612 17446352

[B10] Balakireva M. D. Shevelyov Y. Y. Nurminsky D. I. Livak K. J. Gvozdev V. A. (1992). Structural organization and diversification of Y-linked sequences comprising *Su(Ste)* genes in *Drosophila melanogaster*. Nucleic Acids Res. 20, 3731–3736. 10.1093/nar/20.14.3731 1322529 PMC334025

[B11] Bandyopadhyay M. Arbet S. Bishop C. P. Bidwai A. P. (2016). *Drosophila* protein kinase CK2: genetics, regulatory complexity and emerging roles during development. Pharmaceuticals 10:4. 10.3390/ph10010004 28036067 PMC5374408

[B12] Barbash D. A. Ashburner M. (2003). A novel system of fertility rescue in *Drosophila* hybrids reveals a link between hybrid lethality and female sterility. Genetics 163, 217–226. 12586709 10.1093/genetics/163.1.217 PMC1462402

[B13] Barbash D. A. Roote J. Ashburner M. (2000). The *Drosophila melanogaster* hybrid male rescue gene causes inviability in male and female species hybrids. Genetics 154, 1747–1771. 10747067 10.1093/genetics/154.4.1747 PMC1461041

[B14] Bibby A. C. Litchfield D. W. (2005). The multiple personalities of the regulatory subunit of protein kinase CK2: CK2 dependent and CK2 independent roles reveal a secret identity for CK2beta. Int. J. Biol. Sci. 1, 67–79. 10.7150/ijbs.1.67 15951851 PMC1142214

[B15] Bonet C. Fernández I. Aran X. Bernués J. Giralt E. Azorín F. (2005). The GAGA protein of *Drosophila* is phosphorylated by CK2. J. Mol. Biol. 351, 562–572. 10.1016/j.jmb.2005.06.039 16023138

[B16] Bouazoune K. Brehm A. (2005). dMi-2 chromatin binding and remodeling activities are regulated by dCK2 phosphorylation. J. Biol. Chem. 280, 41912–41920. 10.1074/jbc.M507084200 16223721

[B17] Bozzetti M. P. Massari S. Finelli P. Meggio F. Pinna L. A. Boldyreff B.. (1995). The *Ste* locus, a component of the parasitic *cry-Ste* system of *Drosophila melanogaster*, encodes a protein that forms crystals in primary spermatocytes and mimics properties of the α-subunit of casein kinase 2. Proc. Natl. Acad. Sci. U. S. A. 92, 6067–6071. 10.1073/pnas.92.13.6067 7597082 PMC41643

[B18] Brangwynne C. P. Eckmann C. R. Courson D. S. Rybarska A. Hoege C. Gharakhani J.. (2009). Germline P granules are liquid droplets that localize by controlled dissolution/condensation. Science 324, 1729–1732. 10.1126/science.1172046 19460965

[B19] Brennecke J. Aravin A. A. Stark A. Dus M. Kellis M. Sachidanandam R.. (2007). Discrete small RNA-generating loci as master regulators of transposon activity in Drosophila. Cell 128, 1089–1103. 10.1016/j.cell.2007.01.043 17346786

[B20] Castillo D. M. Barbash D. A. (2017). Moving speciation genetics forward: modern techniques build on foundational studies in *Drosophila*. Genetics 207, 825–842. 10.1534/genetics.116.187120 29097397 PMC5676244

[B21] Chakraborty M. Chang C. Khost D. E. Vedanayagam J. Adrion J. R. Liao Y. (2020). Evolution of genome structure in the *Drosophila simulans* species complex. *bioRxiv [Preprint]*. 10.1101/2020.02.27.968743 PMC7919458 33563718

[B22] Chang C. H. Larracuente A. M. (2019). Heterochromatin-enriched assemblies reveal the sequence and organization of the *Drosophila melanogaster* Y chromosome. Genetics 211, 333–348. 10.1534/genetics.118.301765 30420487 PMC6325706

[B23] Charlesworth B. (2001). Genome analysis: more *Drosophila* Y chromosome genes. Curr. Biol. 11, R182–R184. 10.1016/S0960-9822(01)00089-6 11267888

[B24] Chen P. Kotov A. A. Godneeva B. K. Bazylev S. S. Olenina L. V. Aravin A. A. (2020). piRNA-mediated gene regulation and adaptation to sex-specific transposon expression in *D. melanogaster* male germline. *bioRxiv [Preprint]*. 10.1101/2020.08.25.266585 PMC8168559 33985970

[B25] Conant G. C. Wolfe K. H. (2008). Turning a hobby into a job: how duplicated genes find new functions. Nat. Rev. Genet. 9, 938–950. 10.1038/nrg2482 19015656

[B26] Czech B. Hannon G. J. (2016). One loop to rule them all: the ping-pong cycle and piRNA-guided silencing. Trends Biochem. Sci. 41, 324–337. 10.1016/j.tibs.2015.12.008 26810602 PMC4819955

[B27] Danilevskaya O. N. Kurenova E. V. Pavlova M. N. Bebehov D. V. Link A. J. Koga A.. (1991). He-T family DNA sequences in the Y chromosome of *Drosophila melanogaster* share homology with the X-linked *stellate* genes. Chromosoma 100, 118–124. 10.1007/BF00418245 1672635

[B28] Dobzhansky T. (1936). Studies on hybrid sterility. II. Localization of sterility factors in *Drosophila pseudoobscura* hybrids. Genetics 21, 113–135. 17246786 10.1093/genetics/21.2.113 PMC1208664

[B29] Egorova K. S. Olenkina O. M. Kibanov M. V. Kalmykova A. I. Gvozdev V. A. Olenina L. V. (2009). Genetically derepressed nucleoplasmic stellate protein in spermatocytes of *D. melanogaster* interacts with the catalytic subunit of protein kinase 2 and carries histone-like lysine-methylated mark. J. Mol. Biol. 389, 895–906. 10.1016/j.jmb.2009.04.064 19422836

[B30] Findley S. D. Tamanaha M. Clegg N. J. Ruohola-Baker H. (2003). Maelstrom, a *Drosophila* spindle-class gene, encodes a protein that colocalizes with Vasa and RDE1/AGO1 homolog, Aubergine, in nuage. Development 130, 859–871. 10.1242/dev.00310 12538514

[B31] Fischle W. Tseng B. S. Dormann H. L. Ueberheide B. M. Garcia B. A.. (2005). Regulation of HP1-chromatin binding by histone H3 methylation and phosphorylation. Nature 438, 1116–1122. 10.1038/nature04219 16222246

[B32] Gainetdinov I. Colpan C. Arif A. Cecchini K. Zamore P. D. (2018). A single mechanism of biogenesis, initiated and directed by PIWI proteins, explains piRNA production in most animals. Mol. Cell 71, 775–790. 10.1016/j.molcel.2018.08.007 30193099 PMC6130920

[B33] Haerty W. Singh R. S. (2006). Gene regulation divergence is a major contributor to the evolution of Dobzhansky-Muller incompatibilities between species of *Drosophila*. Mol. Biol. Evol. 23, 1707–1714. 10.1093/molbev/msl033 16757655

[B34] Han B. W. Wang W. Li C. Weng Z. Zamore P. D. (2015). Noncoding RNA. piRNA-guided transposon cleavage initiates Zucchini-dependent, phased piRNA production. Science 348, 817–821. 10.1126/science.aaa1264 25977554 PMC4545291

[B35] Hardy R. W. Lindsley D. L. Livak K. J. Lewis B. Siversten A. V. Joslyn G. L.. (1984). Cytogenetic analysis of a segment of the Y chromosome of *Drosophila melanogaster*. Genetics 107, 591–610. 6430748 10.1093/genetics/107.4.591 PMC1202379

[B36] Hoskins R. A. Smith C. D. Carlson J. W. Carvalho A. B. Halpern A. Kaminker J. S.. (2002). Heterochromatic sequences in a *Drosophila* whole-genome shotgun assembly. Genome Biol. 3:RESEARCH0085. 10.1186/gb-2002-3-12-research0085 12537574 PMC151187

[B37] Huang X. Fejes Tóth K. Aravin A. A. (2017). piRNA biogenesis in *Drosophila melanogaster*. Trends Genet. 33, 882–894. 10.1016/j.tig.2017.09.002 28964526 PMC5773129

[B38] Inoue A. Hyle J. Lechner M. S. Lahti J. M. (2008). Perturbation of HP1 localization and chromatin binding ability causes defects in sister-chromatid cohesion. Mutat. Res. 657, 48–55. 10.1016/j.mrgentox.2008.08.010 18790078

[B39] Kalmykova A. I. Nurminsky D. I. Ryzhov D. V. Shevelyov Y. Y. (2005). Regulated chromatin domain comprising cluster of co-expressed genes in *Drosophila melanogaster*. Nucleic Acids Res. 33, 1435–1444. 10.1093/nar/gki281 15755746 PMC1062873

[B40] Kalmykova A. I. Shevelyov Y. Y. Dobritsa A. A. Gvozdev V. A. (1997). Acquisition and amplification of a testis-expressed autosomal gene, *SSL*, by the *Drosophila* Y chromosome. Proc. Natl. Acad. Sci. U. S. A. 94, 6297–6302. 10.1073/pnas.94.12.6297 9177211 PMC21043

[B41] Kalmykova A. I. Shevelyov Y. Y. Polesskaya O. O. Dobritsa A. A. Evstafieva A. G. Boldyreff B.. (2002). *CK2(beta)tes* gene encodes a testis-specific isoform of the regulatory subunit of casein kinase 2 in *Drosophila melanogaster*. Eur. J. Biochem. 269, 1418–1427. 10.1046/j.1432-1033.2002.02785.x 11874456

[B42] Karandikar U. Anderson S. Mason N. Trott R. L. Bishop C. P. Bidwai A. P. (2003). The *Drosophila SSL* gene is expressed in larvae, pupae, and adults, exhibits sexual dimorphism, and mimics properties of the beta subunit of casein kinase II. Biochem. Biophys. Res. Commun. 301, 941–947. 10.1016/S0006-291X(03)00073-1 12589803

[B43] Kibanov M. V. Egorova K. S. Ryazansky S. S. Sokolova O. A. Kotov A. A. Olenkina O. M.. (2011). A novel organelle, the piNG-body, in the *nuage* of *Drosophila* male germ cells is associated with piRNA-mediated gene silencing. Mol. Biol. Cell. 22, 3410–3419. 10.1091/mbc.e11-02-0168 21775629 PMC3172265

[B44] Kibanov M. V. Kotov A. A. Olenina L. V. (2013). Multicolor fluorescence imaging of whole-mount *Drosophila* testes for studying spermatogenesis. Anal. Biochem. 436, 55–64. 10.1016/j.ab.2013.01.009 23357237

[B45] Kirino Y. Kim N. de Planell-Saguer M. Khandros E. Chiorean S. Klein P. S.. (2009). Arginine methylation of Piwi proteins catalysed by dPRMT5 is required for Ago3 and Aub stability. Nat. Cell Biol. 11, 652–658. 10.1038/ncb1872 19377467 PMC2746449

[B46] Kogan G. L. Epstein V. N. Aravin A. A. Gvozdev V. A. (2000). Molecular evolution of two paralogous tandemly repeated heterochromatic gene clusters linked to the X and Y chromosomes of *Drosophila melanogaster*. Mol. Biol. Evol. 17, 697–702. 10.1093/oxfordjournals.molbev.a026348 10779530

[B47] Kogan G. L. Usakin L. A. Ryazansky S. S. Gvozdev V. A. (2012). Expansion and evolution of the X-linked testis specific multigene families in the *melanogaster* species subgroup. PLoS ONE 7:e37738. 10.1371/journal.pone.0037738 22649555 PMC3359341

[B48] Kotelnikov R. N. Klenov M. S. Rozovsky Y. M. Olenina L. V. Kibanov M. V. Gvozdev V. A. (2009). Peculiarities of piRNA-mediated post-transcriptional silencing of *Stellate* repeats in testes of *Drosophila melanogaster*. Nucleic Acids Res. 37, 3254–3263. 10.1093/nar/gkp167 19321499 PMC2691822

[B49] Kotov A. A. Adashev V. E. Godneeva B. K. Ninova M. Shatskikh A. S. Bazylev S. S.. (2019). piRNA silencing contributes to interspecies hybrid sterility and reproductive isolation in *Drosophila melanogaster*. Nucleic Acids Res. 47, 4255–4271. 10.1093/nar/gkz130 30788506 PMC6486647

[B50] Kouzarides T. (2007). Chromatin modifications and their function. Cell 128, 693–705. 10.1016/j.cell.2007.02.005 17320507

[B51] Le Thomas A. Stuwe E. Li S. Du J. Marinov G. Rozhkov N.. (2014). Transgenerationally inherited piRNAs trigger piRNA biogenesis by changing the chromatin of piRNA clusters and inducing precursor processing. Genes Dev. 28, 1667–1680. 10.1101/gad.245514.114 25085419 PMC4117942

[B52] Lechner M. S. Schultz D. C. Negorev D. Maul G. G. Rauscher F. J. III. (2005). The mammalian heterochromatin protein 1 binds diverse nuclear proteins through a common motif that targets the chromoshadow domain. Biochem. Biophys. Res. Commun. 331, 929–937. 10.1016/j.bbrc.2005.04.016 15882967

[B53] Ledent V. Vervoort M. (2001). The basic helix-loop-helix protein family: comparative genomics and phylogenetic analysis. Genome Res. 11, 754–770. 10.1101/gr.177001 11337472 PMC311049

[B54] Li C. Vagin V. V. Lee S. Xu J. Ma S. Xi H.. (2009). Collapse of germline piRNAs in the absence of Argonaute3 reveals somatic piRNAs in flies. Cell 137, 509–521. 10.1016/j.cell.2009.04.027 19395009 PMC2768572

[B55] Lifschytz E. Hareven D. (1977). Gene expression and the control of spermatid morphogenesis in *Drosophila melanogaster*. Dev. Biol. 58, 276–294. 10.1016/0012-1606(77)90092-6 407115

[B56] Lim A. K. Kai T. (2007). Unique germ-line organelle, *nuage*, functions to repress selfish genetic elements in *Drosophila melanogaster*. Proc. Natl. Acad. Sci. U. S. A. 104, 6714–6719. 10.1073/pnas.0701920104 17428915 PMC1871851

[B57] Lim A. K. Tao L. Kai T. (2009). piRNAs mediate posttranscriptional retroelement silencing and localization to pi-bodies in the *Drosophila* germline. J. Cell Biol. 186, 333–342. 10.1083/jcb.200904063 19651888 PMC2728408

[B58] Livak K. J. (1984). Organization and mapping of a sequence on the *Drosophila melanogaster* X and Y chromosomes that is transcribed during spermatogenesis. Genetics 107, 611–634. 6430749 10.1093/genetics/107.4.611 PMC1202380

[B59] Livak K. J. (1990). Detailed structure of the *Drosophila melanogaster Stellate* genes and their transcripts. Genetics 124, 303–316. 1689686 10.1093/genetics/124.2.303 PMC1203923

[B60] Lyckegaard E. M. Clark A. G. (1989). Ribosomal DNA and *Stellate* gene copy number variation on the Y chromosome of *Drosophila melanogaster*. Proc. Natl. Acad. Sci. U. S. A. 86, 1944–1948. 10.1073/pnas.86.6.1944 2494656 PMC286821

[B61] Malone C. D. Brennecke J. Dus M. Stark A. Mccombie W. R. Sachidanandam R.. (2009). Specialized piRNA pathways act in germline and somatic tissues of the *Drosophila* ovary. Cell 137, 522–535. 10.1016/j.cell.2009.03.040 19395010 PMC2882632

[B62] McKee B. D. Satter M. T. (1996). Structure of the Y chromosomal *Su(Ste)* locus in *Drosophila melanogaster* and evidence for localized recombination among repeats. Genetics 142, 149–161. 8770592 10.1093/genetics/142.1.149 PMC1206943

[B63] Meyer G. F. Hess O. Beermann W. (1961). Phase-specific function structure in spermatocyte nuclei of *Drosophila melanogaster* and their dependence of Y chromosomes. Chromosoma 12, 676–716. 10.1007/BF00328946 14473096

[B64] Michalak P. Noor M. A. (2003). Genome-wide patterns of expression in *Drosophila* pure species and hybrid males. Mol. Biol. Evol. 20, 1070–1076. 10.1093/molbev/msg119 12777520

[B65] Moehring A. J. Teeter K. C. Noor M. A. (2007). Genome-wide patterns of expression in *Drosophila* pure species and hybrid males. II. Examination of multiple-species hybridizations, platforms, and life cycle stages. Mol. Biol. Evol. 24, 137–145. 10.1093/molbev/msl142 17032727

[B66] Mohn F. Handler D. Brennecke J. (2015). Noncoding RNA. piRNA-guided slicing specifies transcripts for Zucchini-dependent, phased piRNA biogenesis. Science 348, 812–817. 10.1126/science.aaa1039 25977553 PMC4988486

[B67] Muller H. J. (1940). Bearings of the *Drosophila* work on systematics, in The New Systematics, ed J. Huxley (Oxford: Clarendon Press), 185–268.

[B68] Muller H. J. Pontecorvo G. (1942). Recessive genes causing interspecific sterility and other disharmonies between *Drosophila melanogaster* and *simulans. Genetics* 27:157.

[B69] Ninova M. Fejes Tóth K. Aravin A. A. (2019). The control of gene expression and cell identity by H3K9 trimethylation. Development 146:dev181180. 10.1242/dev.181180 31540910 PMC6803365

[B70] Nishida K. M. Okada T. N. Kawamura T. Mituyama T. Kawamura Y. Inagaki S.. (2009). Functional involvement of Tudor and dPRMT5 in the piRNA processing pathway in *Drosophila* germlines. EMBO J. 28, 3820–3831. 10.1038/emboj.2009.365 19959991 PMC2797066

[B71] Nishida K. M. Saito K. Mori T. Kawamura Y. Nagami-Okada T. Inagaki S.. (2007). Gene silencing mechanisms mediated by Aubergine piRNA complexes in *Drosophila* male gonad. RNA 13, 1911–1922. 10.1261/rna.744307 17872506 PMC2040086

[B72] Nosov G. A. Kibanov M. V. Olenina L. V. (2014). Dynamic properties of germinal granule ping-body in the testes of *Drosophila melanogaster*. Mol. Biol. 48, 805–813. 10.1134/S0026893314050112 25842866

[B73] Nott T. J. Petsalaki E. Farber P. Jervis D. Fussner E. Plochowietz A.. (2015). Phase transition of a disordered *nuage* protein generates environmentally responsive membraneless organelles. Mol. Cell 57, 936–947. 10.1016/j.molcel.2015.01.013 25747659 PMC4352761

[B74] Olenkina O. M. Egorova K. S. Aravin A. A. Naumova N. M. Gvozdev V. A. Olenina L. V. (2012b). Mapping of *cis*-regulatory sites in the promoter of testis-specific *Stellate* genes of *Drosophila melanogaster*. Biochem. Mosc. 77, 1285–1293. 10.1134/S0006297912110077 23240566

[B75] Olenkina O. M. Egorova K. S. Kibanov M. V. Gervaziev Y. V. Gvozdev V. A. Olenina L. V. (2012a). Promoter contribution to the testis-specific expression of *Stellate* gene family in *Drosophila melanogaster*. Gene 499, 143–153. 10.1016/j.gene.2012.03.023 22425977

[B76] Orr H. A. (2005). The genetic basis of reproductive isolation: insights from *Drosophila. Proc. Natl. Acad. Sci. U. S. A*. 102, 6522–6526. 10.1073/pnas.0501893102 15851676 PMC1131866

[B77] Ozata D. M. Gainetdinov I. Zoch A. O'Carroll D. Zamore P. D. (2019). PIWI-interacting RNAs: small RNAs with big functions. Nat. Rev. Genet. 20, 89–108. 10.1038/s41576-018-0073-3 30446728

[B78] Özata D. M. Yu T. Mou H. Gainetdinov I. Colpan C. Cecchini K.. (2020). Evolutionarily conserved pachytene piRNA loci are highly divergent among modern humans. Nat. Ecol. Evol. 4, 156–168. 10.1038/s41559-019-1065-1 31900453 PMC6961462

[B79] Palumbo G. Bonaccorsi S. Robbins L. G. Pimpinelli S. (1994). Genetic analysis of *Stellate* elements of *Drosophila melanogaster*. Genetics 138, 1181–1197. 7896100 10.1093/genetics/138.4.1181 PMC1206257

[B80] Pane A. Wehr K. Schüpbach T. (2007). *zucchini* and *squash* encode two putative nucleases required for rasiRNA production in the *Drosophila* germline. Dev. Cell 12, 851–862. 10.1016/j.devcel.2007.03.022 17543859 PMC1945814

[B81] Patil V. S. Kai T. (2010). Repression of retroelements in *Drosophila* germline via piRNA pathway by the Tudor domain protein Tejas. Curr. Biol. 20, 724–730. 10.1016/j.cub.2010.02.046 20362446

[B82] Pinna L. A. (2002). Protein kinase CK2: a challenge to canons. J. Cell Sci. 115, 3873–3878. 10.1242/jcs.00074 12244125

[B83] Rathert P. Dhayalan A. Murakami M. Zhang X. Tamas R. Jurkowska R.. (2008). Protein lysine methyltransferase G9a acts on non-histone targets. Nat. Chem. Biol. 4, 344–346. 10.1038/nchembio.88 18438403 PMC2696268

[B84] Rojas-Ríos P. Chartier A. Pierson S. Simonelig M. (2017). Aubergine and piRNAs promote germline stem cell self-renewal by repressing the proto-oncogene Cbl. EMBO J. 36, 3194–3211. 10.15252/embj.201797259 29030484 PMC5666619

[B85] Rojas-Ríos P. Simonelig M. (2018). piRNAs and PIWI proteins: regulators of gene expression in development and stem cells. Development 145:dev161786. 10.1242/dev.161786 30194260

[B86] Russo C. A. Takezaki N. Nei M. (1995). Molecular phylogeny and divergence times of drosophilid species. Mol. Biol. Evol. 12, 391–404. 7739381 10.1093/oxfordjournals.molbev.a040214

[B87] Ryazansky S. S. Kotov A. A. Kibanov M. V. Akulenko N. V. Korbut A. P. Lavrov S. A.. (2016). RNA helicase Spn-E is required to maintain Aub and AGO3 protein levels for piRNA silencing in the germline of *Drosophila*. Eur. J. Cell Biol. 95, 311–322. 10.1016/j.ejcb.2016.06.001 27320195

[B88] Schotta G. Ebert A. Krauss V. Fischer A. Hoffmann J. Rea S.. (2002). Central role of *Drosophila* SU(VAR)3-9 in histone H3-K9 methylation and heterochromatic gene silencing. EMBO J. 21, 1121–1131. 10.1093/emboj/21.5.1121 11867540 PMC125909

[B89] Schultz D. C. Ayyanathan K. Negorev D. Maul G. G. Rauscher F. J. III. (2002). SETDB1: a novel KAP-1-associated histone H3, lysine 9-specific methyltransferase that contributes to HP1-mediated silencing of euchromatic genes by KRAB zinc-finger proteins. Genes Dev. 16, 919–932. 10.1101/gad.973302 11959841 PMC152359

[B90] Senti K. A. Jurczak D. Sachidanandam R. Brennecke J. (2015). piRNA-guided slicing of transposon transcripts enforces their transcriptional silencing via specifying the nuclear piRNA repertoire. Genes Dev. 29, 1747–1762. 10.1101/gad.267252.115 26302790 PMC4561483

[B91] Shevelyov Y. Y. (1992). Copies of a *Stellate* gene variant are located in the X heterochromatin of *Drosophila melanogaster* and are probably expressed. Genetics 132, 1033–1037. 1459425 10.1093/genetics/132.4.1033 PMC1205225

[B92] Snee M. J. Macdonald P. M. (2004). Live imaging of *nuage* and polar granules: evidence against a precursor-product relationship and a novel role for Oskar in stabilization of polar granule components. J. Cell Sci. 117, 2109–2120. 10.1242/jcs.01059 15090597

[B93] Tachibana M. Sugimoto K. Fukushima T. Shinkai Y. (2001). SET Domain-containing protein, G9a, is a novel lysine-preferring mammalian histone methyltransferase with hyperactivity and specific selectivity to lysines 9 and 27 of histone H3. J. Biol. Chem. 276, 25309–25317. 10.1074/jbc.M101914200 11316813

[B94] Tamura K. Subramanian S. Kumar S. (2004). Temporal patterns of fruit fly (*Drosophila*) evolution revealed by mutation clocks. Mol. Biol. Evol. 21, 36–44. 10.1093/molbev/msg236 12949132

[B95] Tulin A. V. Kogan G. L. Filipp D. Balakireva M. D. Gvozdev V. A. (1997). Heterochromatic *Stellate* gene cluster in *Drosophila melanogaster*: structure and molecular evolution. Genetics 146, 253–262. 9136015 10.1093/genetics/146.1.253 PMC1207940

[B96] Usakin L. A. Kogan G. L. Kalmykova A. I. Gvozdev V. A. (2005). An alien promoter capture as a primary step of the evolution of testes-expressed repeats in the *Drosophila melanogaster* genome. Mol. Biol. Evol. 22, 1555–1560. 10.1093/molbev/msi147 15829619

[B97] Vagin V. V. Sigova A. Li C. Seitz H. Gvozdev V. Zamore P. D. (2006). A distinct small RNA pathway silences selfish genetic elements in the germline. Science 313, 320–324. 10.1126/science.1129333 16809489

[B98] Vermaak D. Malik H. S. (2009). Multiple roles for heterochromatin protein 1 genes in *Drosophila*. Annu. Rev. Genet. 43, 467–492. 10.1146/annurev-genet-102108-134802 19919324

[B99] Wang W. Han B. W. Tipping C. Ge D. T. Zhang Z. Weng Z.. (2015). Slicing and binding by Ago3 or Aub trigger piwi-bound piRNA production by distinct mechanisms. Mol. Cell 59, 819–830. 10.1016/j.molcel.2015.08.007 26340424 PMC4560842

[B100] Webster A. Li S. Hur J. K. Wachsmuth M. Bois J. S. Perkins E. M.. (2015). Aub and Ago3 are recruited to nuage through two mechanisms to form a ping-pong complex assembled by Krimper. Mol. Cell 59, 564–575. 10.1016/j.molcel.2015.07.017 26295961 PMC4545750

[B101] Wei K. H. Clark A. G. Barbash D. A. (2014). Limited gene misregulation is exacerbated by allele-specific upregulation in lethal hybrids between *Drosophila melanogaster* and *Drosophila simulans*. Mol. Biol. Evol. 31, 1767–1778. 10.1093/molbev/msu127 24723419 PMC4069615

[B102] Xiol J. Spinelli P. Laussmann M. A. Homolka D. Yang Z. Cora E.. (2014). RNA clamping by Vasa assembles a piRNA amplifier complex on transposon transcripts. Cell 157, 1698–1711. 10.1016/j.cell.2014.05.018 24910301

[B103] Zhang F. Wang J. Xu J. Zhang Z. Koppetsch B. S. Schultz N.. (2012). UAP56 couples piRNA clusters to the perinuclear transposon silencing machinery. Cell 151, 871–884. 10.1016/j.cell.2012.09.040 23141543 PMC3499805

[B104] Zhang Z. Xu J. Koppetsch B. S. Wang J. Tipping C. Ma S.. (2011). Heterotypic piRNA Ping-Pong requires qin, a protein with both E3 ligase and Tudor domains. Mol. Cell 44, 572–584. 10.1016/j.molcel.2011.10.011 22099305 PMC3236501

[B105] Zhao T. Eissenberg J. C. (1999). Phosphorylation of heterochromatin protein 1 by casein kinase II is required for efficient heterochromatin binding in *Drosophila*. J. Biol. Chem. 274, 15095–15100. 10.1074/jbc.274.21.15095 10329715

